# Quantification of Cardiac Kinetic Energy and Its Changes During Transmural Myocardial Infarction Assessed by Multi-Dimensional Seismocardiography

**DOI:** 10.3389/fcvm.2021.603319

**Published:** 2021-03-08

**Authors:** Sofia Morra, Lorenzo Pitisci, Fuhong Su, Amin Hossein, Jérémy Rabineau, Judith Racape, Damien Gorlier, Antoine Herpain, Pierre-François Migeotte, Jacques Creteur, Philippe van de Borne

**Affiliations:** ^1^Department of Cardiology, Erasme Hospital, Université Libre de Bruxelles, Brussels, Belgium; ^2^Experimental Laboratory of Intensive Care, Erasme Hospital, Université Libre de Bruxelles, Brussels, Belgium; ^3^Laboratory of Physic and Physiology (LPHYS), Université Libre de Bruxelles, Brussels, Belgium; ^4^Research Center in Epidemiology, Biostatistics and Clinical Research, School of Public Health, Université Libre de Bruxelles (ULB), Brussels, Belgium; ^5^Department of Intensive Care, Erasme Hospital, Université Libre de Bruxelles, Brussels, Belgium

**Keywords:** seismocardiography, kinetic energy, acute myocardial infarction, animal model for acute coronary syndrome, cardiac monitoring

## Abstract

**Introduction:** Seismocardiography (SCG) records cardiac and blood-induced motions transmitted to the chest surface as vibratory phenomena. Evidences demonstrate that acute myocardial ischemia (AMI) profoundly affects the SCG signals. Multidimensional SCG records cardiac vibrations in linear and rotational dimensions, and scalar parameters of kinetic energy can be computed. We speculate that AMI and revascularization profoundly modify cardiac kinetic energy as recorded by SCG.

**Methods:** Under general anesthesia, 21 swine underwent 90 min of myocardial ischemia induced by percutaneous sub-occlusion of the proximal left anterior descending (LAD) coronary artery and subsequent revascularization. Invasive hemodynamic parameters were continuously recorded. SCG was recorded during baseline, immediately and 80 min after LAD sub-occlusion, and immediately and 60 min after LAD reperfusion. *i*K was automatically computed for each cardiac cycle (*iK*^*CC*^) in linear (*iK*_*Lin*_) and rotational (*iK*_*Rot*_) dimensions. *i*K was calculated as well during systole and diastole (*iK*^*Sys*^ and *iK*^*Dia*^, respectively). Echocardiography was performed at baseline and after revascularization, and the left ventricle ejection fraction (LVEF) along with regional left ventricle (LV) wall abnormalities were evaluated.

**Results:** Upon LAD sub-occlusion, 77% of STEMI and 24% of NSTEMI were observed. Compared to baseline, troponins increased from 13.0 (6.5; 21.3) ng/dl to 170.5 (102.5; 475.0) ng/dl, and LVEF dropped from 65.0 ± 0.0 to 30.6 ± 5.7% at the end of revascularization (both *p* < 0.0001). Regional LV wall abnormalities were observed as follows: anterior MI, 17.6% (three out of 17); septal MI, 5.8% (one out of 17); antero-septal MI, 47.1% (eight out of 17); and infero-septal MI, 29.4% (five out of 17). In the linear dimension, ${iK}_{Lin}^{CC}$, ${iK}_{Lin}^{Sys}$, and ${iK}_{Lin}^{Dia}$ dropped by 43, 52, and 53%, respectively (*p* < 0.0001, *p* < 0.0001, and *p* = 0.03, respectively) from baseline to the end of reperfusion. In the rotational dimension, ${iK}_{Rot}^{CC}$ and ${iK}_{Rot}^{Sys}$ dropped by 30 and 36%, respectively (*p* = 0.0006 and *p* < 0.0001, respectively), but ${iK}_{Rot}^{Dia}$ did not change (*p* = 0.41). All the hemodynamic parameters, except the pulmonary artery pulse pressure, were significantly correlated with the parameters of *iK*, except for the diastolic component.

**Conclusions:** In this very context of experimental AMI with acute LV regional dysfunction and no concomitant AMI-related heart valve disease, linear and rotational *i*K parameters, in particular, systolic ones, provide reliable information on LV contractile dysfunction and its effects on the downstream circulation. Multidimensional SCG may provide information on the cardiac contractile status expressed in terms of *i*K during AMI and reperfusion. This automatic system may empower health care providers and patients to remotely monitor cardiovascular status in the near future.

## Introduction

Ballistocardiography (BCG) and seismocardiography (SCG) record the micro-vibrations produced rhythmically by velocities and accelerations of blood mass flowing across cardiac chambers and main vessels as a consequence of cardiac mass contraction, with micro-accelerometers and gyroscopes placed on the body surface (1–3). There is growing evidence that BCG and SCG may provide additional relevant information on cardiovascular status beyond those already acquired by means of universally accepted current diagnostic devices. Indeed BCG and SCG reliably estimate stroke volume (SV) and cardiac output (CO) (2, 4), myocardial contractility expressed as d*P*/d*t*_max_ in animal models (5), as well as the clinical status of heart failure patients (6).

As a result, this evidence fuels the curiosity of scientific and medical researchers who actively inquire on the potential of BCG and SCG signals to assess cardiovascular mechanical changes during acute myocardial infarction (AMI) (5, 7–9). Indeed the BCG and SCG signals profoundly change during AMI, and, according to previous studies, metrics secured from it enable the identification of an impairment of regional myocardial contraction due to acute ischemia with specificity of 80% (7) to 92% (9) and sensitivity of 89% (7) to 94% (9). When combined with the electrocardiogram (ECG), the SCG empowers the capability of detection of coronary artery disease during an exercise stress test, yielding a positive predictive value of 88% and a negative predictive value of 80% (10).

Recently, a multi-dimensional BCG combined with a multi-dimensional SCG, called kinocardiograph (KCG), has been introduced and, differently from many previous devices which record signals only in one dimension, the KCG records both three-dimensional (3D) linear acceleration and 3D angular velocity signals by means of linear and rotational channels (2, 11). Using specific algorithms, kinetic energy and its temporal integral (*iK*) can be computed from the BCG and SCG waveforms as scalar parameters, both in a linear (*iK*_*Lin*_) and in a rotational (*iK*_*Rot*_) dimension (2, 12).

Three-axes linear micro-accelerometers have already been shown useful in the early detection of experimental AMI (7–9, 13, 14). However, whether non-invasive accelerometers and gyroscopes recording signals in multiple (linear and rotational) dimensions can be affected by hemodynamic changes during acute myocardial infarction and reperfusion is not known. Recently, non-invasive techniques based on micro-accelerometers and gyroscopes exploring rotational velocities and accelerations produced by heart contraction have been introduced (15–17): rotational velocities measured using non-invasive tri-axial gyroscopes provide information on several mechanical events occurring during a contractile cycle as compared to echocardiography (16). The rotational kinetic energy obtained from tri-axial gyroscopes can accurately identify the first and the second peak of the SCG (15). Rotational, rather than linear, kinetic energy accounts for about 70% of the total cardiac energy produced during a contractile cycle, and it significantly drops after prolonged cardiac deconditioning, mainly due to a decrease in the rotational twist of the LV (18). Measuring the rotational accelerations and kinetic energy may contribute to a more in-depth and global analysis of cardiac function seen through the windows of micro-accelerations since the rotational motion of the heart along its longitudinal axis is crucial in assuring its pumping function (19).

Using an animal model for AMI, the present investigation aims (1) to track modifications of linear and rotational *i*K computed from the accelerations signals of non-invasive and multidimensional SCG during coronary artery sub-occlusion, (2) to follow these changes during the reperfusion period, and (3) to study the association of linear and rotational *i*K with invasive hemodynamic parameters. The hypothesis tested is that experimental AMI and reperfusion profoundly alter multi-dimensional SCG signals and its derived scalar parameters.

## Materials and Methods

### Study Protocol

The present study was approved by the Institutional Ethics Committee on Animal Welfare from the Faculty of Medicine of the Université Libre de Bruxelles (ULB, Brussels, Belgium) (acceptance number: 654N). Animal care and handling were in accordance with the National Institute of Health Guidelines.

The procedure consisted in the proximal left anterior descending coronary artery (LAD) sub-occlusion by means of angioplasty semi-compliant balloon inflation for 90 min, followed by deflation and subsequent reperfusion (RE) for 60 min. A 3-min-length SCG was recorded during the steady state (baseline, BSL) preceding the LAD sub-occlusion and then at different time points during sub-occlusion and reperfusion, specifically at the onset of LAD sub-occlusion (AMI_t0_) at 80 minutes of AMI (AMI_t80_), at the onset of RE (RE_t0_), and at 60 min of RE (RE_t60_). Each record was remotely acquired with a tablet and sent *via* Bluetooth to the main server for further signal processing. To evaluate the amount of myocardial necrosis, the authors measured the serum troponin levels at the onset of LAD sub-occlusion and after revascularization: the difference between troponins measured 5 h after RE and troponins measured at the onset of LAD sub-occlusion was named Delta (Δ) troponin. Echocardiography was performed at baseline and after revascularization, and the left ventricle ejection fraction (LVEF) along with regional left ventricle (LV) wall abnormalities were evaluated by a trained operator.

### Animal Preparation and Experimental Procedure

The animals have been put on fasting for 18 h before the experiment was started, with unrestricted access to water. Twenty-one 50-kg crossbreed Landrace/Large White adult swine, of either sex, were premedicated with intramuscular neck injection of 5 mg/kg azaperone and 1.5 mg/kg midazolam. A 14-G peripheral venous line was placed into an ear vein to provide vascular access, and a 4.5-Fr arterial catheter (Leader-Cath, Vygon, France) was inserted in the left common femoral artery for invasive arterial pressure monitoring and blood sample collection. A three-lead surface ECG was connected to the hemodynamic monitoring display (SC9000, Siemens, Germany). The animals underwent endotracheal intubation following induction of anesthesia with an intravenous injection of 3 μg/kg sufentanil, 1 mg/kg propofol, and 0.5 mg/kg of rocuronium. A central venous access for drug infusion was obtained via a three-lumens central venous line inserted into the right external jugular vein (Edwards LifeSciences^©^, California, USA). General anesthesia and analgesia were achieved using continuous inhalation of 1.8 to 2.5% sevoflurane of minimal alveolar concentration (MAC) and continuous infusion of sufentanil 1 to 4 μg/kg/h, adapted according to the response to painful stimulations, in association with 1 to 2 mg/kg/h rocuronium continuous infusion to avoid shivering.

Sevoflurane is the most popular volatile agent used to induce general anesthesia, thanks to its safety profile (20, 21): it has low myocardial depressant effect (22); it does not alter the A–H interval, His-Purkinje conduction time (H–V interval), and ventricular conduction time (H–S interval) (23). It is associated with higher hemodynamic stability and fewer arrhythmic events compared to other volatile agents (24). Since it has no effect on the cardiac conduction system, sevoflurane can also be used in cardiac electrophysiological procedure (25). Additionally, at clinical concentrations of this drug, despite the reduction of peripheral vascular resistance, the cardiac output is preserved (26, 27), as well as coronary blood flow (21).

Mechanical ventilation was performed in a volume-controlled mode (Primus®, Draëger, Germany) with tidal volume of 8 ml/kg and a positive end-expiratory pressure set at 5 cm H_2_O.

A 7 Fr introducer was inserted into the left external jugular vein, and a pulmonary artery catheter (CCO; Edwards LifeSciences^©^, California, USA) was advanced in a pulmonary artery for continuous cardiac output (CO), right heart pressures, and mixed venous oxygen saturation (SVO_2_) monitoring. A 5 Fr introducer (Terumo Corporation, Japan) was inserted into the right internal carotid artery, and a coronary guide catheter (Sherpa JL4™, Medtronic, Belgium) was positioned into the left coronary ostium under fluoroscopic guidance with iodinate contrast media angiogram (Xenetix 350®, Guerbet, France). Through this latter step and after an intracoronary bolus of 200 μg dinitrate isosorbide to prevent coronary spasm, pressure and a Doppler flow wires (ComboWire®, Volcano Corporation, Belgium) were placed distally into the mid LAD. Two 5 Fr introducers (Terumo Corporation, Japan) were inserted into the left carotid artery and left femoral artery, where high-fidelity left ventricular pressure—volume catheter (Transonic®, France) and aortic arch catheter (Transonic®, France) were placed.

ECG, pressure and volume signal, CO, and respiratory rate were recorded using a data acquisition software (Notocord-HEM™, France), allowing subsequent offline analysis. The animals were administrated with 300 mg amiodarone, followed by continuous infusions of 900 mg/24 h and 7,500 units of unfractionated heparin, followed by a continuous infusion of 2,000 units/h.

A semi-compliant angioplasty balloon (Trek, Abbott, Belgium) was inserted over the wire into the proximal LAD and was inflated to reduce coronary flow by 60% of the baseline value for 90 min. After 90 min of ischemia, 200 mg of aspirin was administrated intravenously, and the balloon was deflated, allowing reperfusion to occur according to the best current clinical managing of ACS (28). Once the balloon was deflated, the effectiveness of reperfusion was confirmed by the recovery of intracoronary blood flow velocity. Three swine died, during the procedure, from refractory ventricular arrythmias, which occurred within the first 30 min from coronary occlusion.

### Sham Group

A sham group of another experimental procedure (Ethical Committee acceptance number: 641N), following the same protocol of general anesthesia and instrumentation of the animal, was used as a reference to rule out the possible depressant effect of general anesthesia on the hemodynamic parameters. This sham group was composed of three crossbreed Landrace/Large White adult swine (weight: 41, 31, and 46 kg), all undergoing the same general anesthesia protocol and instrumentation that we used in the present investigation. The hemodynamic parameters of each animal were followed at three different timepoints: during BSL, at 2 h (T1), and at 4 h (T2) of steady state, while no intervention was realized. These data show a reduction by ±5 mmHg of mean arterial pressure, concomitant to the experimental setting (Supplementary Table 1). Additionally, no arrhythmic events were observed.

Since the results from a sham group were already available in our laboratory, the local ethical committee for animal care considered it unnecessary to add a sham group in the present investigation.

### Accelerometric Signal Acquisition and Processing

The KCG consisted of two modules, each containing MEMS accelerometers and gyroscope sensors (LSM6DSL, STMicroelectronics). One module was placed over the sternum to record local precordial vibrations (SCG); the other one was placed immediately below and externally to the left iliac crest to record one-lead ECG signal. The device was controlled remotely with a tablet connected *via* Bluetooth and collected a one-lead ECG and a linear (Lin) and a rotational (Rot) three-axes SCG. Details about this methodology have been described previously (2, 12). Observations from unpublished results demonstrate that SCG measurements are reliable and reproducible using different sensors and that the metrics of linear and rotational *i*K are comparable.

Assuming that the cardiovascular system equates a Newtonian system, scalar metrics can be obtained from velocity and acceleration signals measured with the SCG in the linear and rotational dimensions and transmitted to the body surface as vibratory phenomena. The height and weight of the animal are used to assess inertial parameters. Knowing the acceleration of an object with a given mass *m* and the vector force ($\vec{F}$), the kinetic energy (*K*) can be calculated according to Equations (1) and (2) for the linear components and to Equations (3) and (4) for the rotational components.

(1) $$\begin{array}{l} \vec{F}\left(t\right)=m\;\vec{a}\left(t\right) \end{array}$$

(2) $$\begin{array}{l} K_{Lin}\left(t\right)=\frac{1}{2}m\left(v_{x}^{2}\left(t\right)+\;v_{y}^{2}\left(t\right)+\;v_{z}^{2}\left(t\right)\right) \end{array}$$

where *m* is the mass of the object, *K*_Lin_ is the linear kinetic energy, *v*_*x*_, *v*_*y*_, and *v*_*z*_ are components of the measured velocity vector $\vec{v}$, and $\vec{F}$ is the force vector.

For the rotational components, the scalar metrics are calculated according to Equations (3) and (4).

(3) $$\begin{array}{l} \vec{\tau }\left(t\right)=I.\;\vec{\alpha }\left(t\right) \end{array}$$

(4) $$\begin{array}{l} K_{Rot}\left(t\right)=\frac{1}{2}\left(I_{xx}\omega_{x}^{2}\left(t\right)+\;I_{yy}\omega_{y}^{2}\left(t\right)+\;I_{zz}\omega_{zz}^{2}\left(t\right)\right) \end{array}$$

where $\vec{\tau }$ is the torque of force, *I* is the momentum of inertia of the object, $\vec{\alpha }$ is the angular acceleration, *K*_Rot_ is the rotational kinetic energy, *I*_*xx*_, *I*_*yy*_, and *I*_*zz*_ are the orthogonal components of the momentum of inertia *I* of the object, and ω_*x*_, ω_*y*_, and ω_*z*_ are the components of the measured angular velocity $\vec{\omega }$.

The time integral of *K*_Lin_ and *K*_Rot_ over the cardiac cycle (CC) was computed for the SCG as in Equations (5) and (6).

(5) $$\begin{array}{c} iK_{Lin}=\int_{CC}K_{\text{Lin}}(t).dt. \end{array}$$

(6) $$\begin{array}{c} iK_{Rot}=\int_{CC}K_{\text{Rot}}(t).dt. \end{array}$$

Data were acquired at BSL, at AMI_t0−t80_, and at RE_t0−t60_ and then exported and analyzed offline using a toolbox developed in MatLab version 9.5 R2018b (Mathworks®). The operator was selecting a 60-s-width artefact-free temporal window of consecutive beats. The beats were automatically identified based on the automatic identification of the peak ECG-R wave. Ensemble averaging (EA) on all beats over the selected time period was performed, and the scalar parameters of *iK*_*Lin*_ and *iK*_*Rot*_ were automatically computed. This method of sampling and averaging generated an averaged SCG signal which best fits the shape of a cardiac cycle. Additionally, EA was used to partially remove motion artifacts from the signals.

The P, Q, R, S, and T waves on the ECG were automatically identified and used as reference points for the identification of the electrical cardiac cycle. The sum of QRS and ST segments identifies the systolic phase (Sys) of the cardiac cycle; the sum of the TP' segment (the period from the T wave of the current beat *N* to the P wave of the next beat *N* + 1) with the P'Q' segment (the period from the P wave of the beat *N* + 1 to the Q wave of the beat *N* + 1) identifies the diastole (Dia) of the cardiac cycle. The sum of PQ, QRS, ST, and TP' defined a whole CC. One record had to be ruled out from final analysis because of technical failure during the signal processing.

Several factors can contaminate the BCG and SCG signals, such as respiration, involuntary movements, and cough. To reduce contamination signals from artifacts, an automatic outlier detection was applied on beats that would generate too large energies, possibly due to the involuntary movement of the subject such as coughing or deglutition or movements of the extremities. If the *i*K of a heartbeat was higher than five times the median of the respective kinetic energy of the five previous beats, the *i*K of the concerned heartbeat was considered as compromised by a motion artefact and classified as abnormal.

Respiration might influence the BCG and SCG signals in three different ways: by producing a wandering of the baseline as a result of chest movement, by modifying the amplitude of SCG due to intra-thoracic pressure variation, and through the induced RR interval changes during the respiratory cycle. To avoid contamination signal from respiratory movement, a high-pass filter was applied to the signals.

### Statistical Analysis

Statistical analysis was performed using STATACorp® for Windows. GraphPad PRISM® version 5.01 and MatLab version 9.5 R2018b (Mathworks Inc.®) were used for graphing figures on Windows.

Normality of data distribution was assessed graphically and by using the Kolmogorov–Smirnov test. According to the distribution, data were expressed as mean ± standard deviation (±SD) if normally distributed or as median and interquartile range if not [*P*_25_-*P*_75_].

To evaluate the effect of AMI and reperfusion on SCG signals, a generalized mixed model was used, taking time as the fixed factor, followed by multiple comparison whenever a significant effect was found. Bonferroni's correction was applied to account for multiple comparisons.

The pulse pressures of LV, aortic, femoral, and pulmonary artery pressures were calculated as the difference between systolic and diastolic pressures (29). Generalized linear mixed model was used to associate the pulse pressures and CO with the parameters of *iK*.

Spearman's rank correlation was used to assess the association of *iK* parameters with Δ troponins and the LVEF. Correlations were calculated between *i*K parameters and LVEF computed at the end of the procedure (RE_t60_).

*P*-values <0.05 were considered as statistically significant.

## Results

Upon LAD sub-occlusion, 77% (thirteen out of 17) of STEMI and 24% (four out of 17) of NSTEMI were observed.

Compared to baseline, troponins increased from 13.0 (6.5; 21.3) ng/dl to 170.5 (102.5; 475.0) ng/dl and LVEF dropped from 65.0 to 30.6 ± 5.7% at the end of revascularization (both *p* < 0.0001). Regional LV wall abnormalities were observed as follows: anterior MI, 17.6% (three out of 17); septal MI, 5.8% (one out of 17); antero-septal MI, 47.1% (eight out of 17); and infero-septal, 29.4% (five out of 17). The animals did not disclose valve diseases at baseline, and there were no AMI-related valve diseases throughout the study.

Modifications of heart rate (HR), CO, systolic and diastolic LV pressures (PLV Sys and PLV Dia, respectively), systolic and diastolic aortic pressures (PAo Sys and PAo Dia, respectively), systolic and diastolic femoral pressures (Pfem Sys and Pfem Dia, respectively), and systolic and diastolic pulmonary artery pressures (PAP Sys and PAP Dia, respectively) during LAD sub-occlusion and reperfusion are reported in Table 1.

**Table 1:** Modification of hemodynamic parameters during left anterior descending occlusion and reperfusion.

**Time**	**HR (bpm)**	**CO (L/min)**	**PLV Sys (mmHg)**	**PLV Dia (mmHg)**	**PAo Sys (mmHg)**	**PAo Dia (mmHg)**	**Pfem Sys (mmHg)**	**Pfem Dia (mmHg)**	**PAP Sys (mmHg)**	**PAP Dia (mmHg)**
BSL	74 ± 14	5.3 ± 1.1	96 ± 8	3 ± 6	94 ± 6	55 ± 6	98 ± 20	52 ± 14	36 ± 5	16 ± 4
AMI_t0_	77 ± 14	5.1 ± 0.8	80 ± 9^*^	5 ± 4	78 ± 10^*^	47 ± 10	80 ± 17^‡^	47 ± 14	29 ± 6	14 ± 4
AMI_t80_	85 ± 18	4.7 ± 0.85^†^	80 ± 6^*^	5 ± 4	79 ± 7^*^	50 ± 8	79 ± 22^*^	46 ± 16	33 ± 4	16 ± 3
RE_t0_	87 ± 18	4.7 ± 0.64^†^	81 ± 7^*^	5 ± 4	80 ± 8^*^	49 ± 9	83 ± 22^‡^	47 ± 17	33 ± 4	16 ± 4
RE_t60_	89 ± 17^*^^a^	4.4 ± 0.96^‡^	82 ± 7^*^	5 ± 3	80 ± 9^*^	49 ± 11	86 ± 18	50 ± 11	33 ± 11	15 ± 5
PALL value	0.0001	0.0001	0.005	ns	0.005	ns	0.0001	ns	ns	ns

*BSL, baseline; AMI*_*t*0−−*t*80_*, acute myocardial infarction at t0 and t80, respectively; RE*_*t*0−−*t*60_*, reperfusion at t0 and t60, respectively; HR, heart rate; PLV Sys and PLV Dia, systolic and diastolic LV pressures, respectively; PAo Sys and PAo Dia, systolic and diastolic aortic pressures, respectively; Pfem Sys and Pfem Dia, systolic and diastolic femoral pressures, respectively; PAP Sys and PAP Dia, systolic and diastolic pulmonary arterial pressures, respectively*.

*Results from multiple-comparison analysis account for comparison of the different timepoints against BSL. Data are presented as mean* ± *SD*.

* *p* < *0.05*;

† *p* < *0.01*;

‡ *p* < *0.0001*.

a *Comparison is significant against AMIt0*.

**Figure 1:**
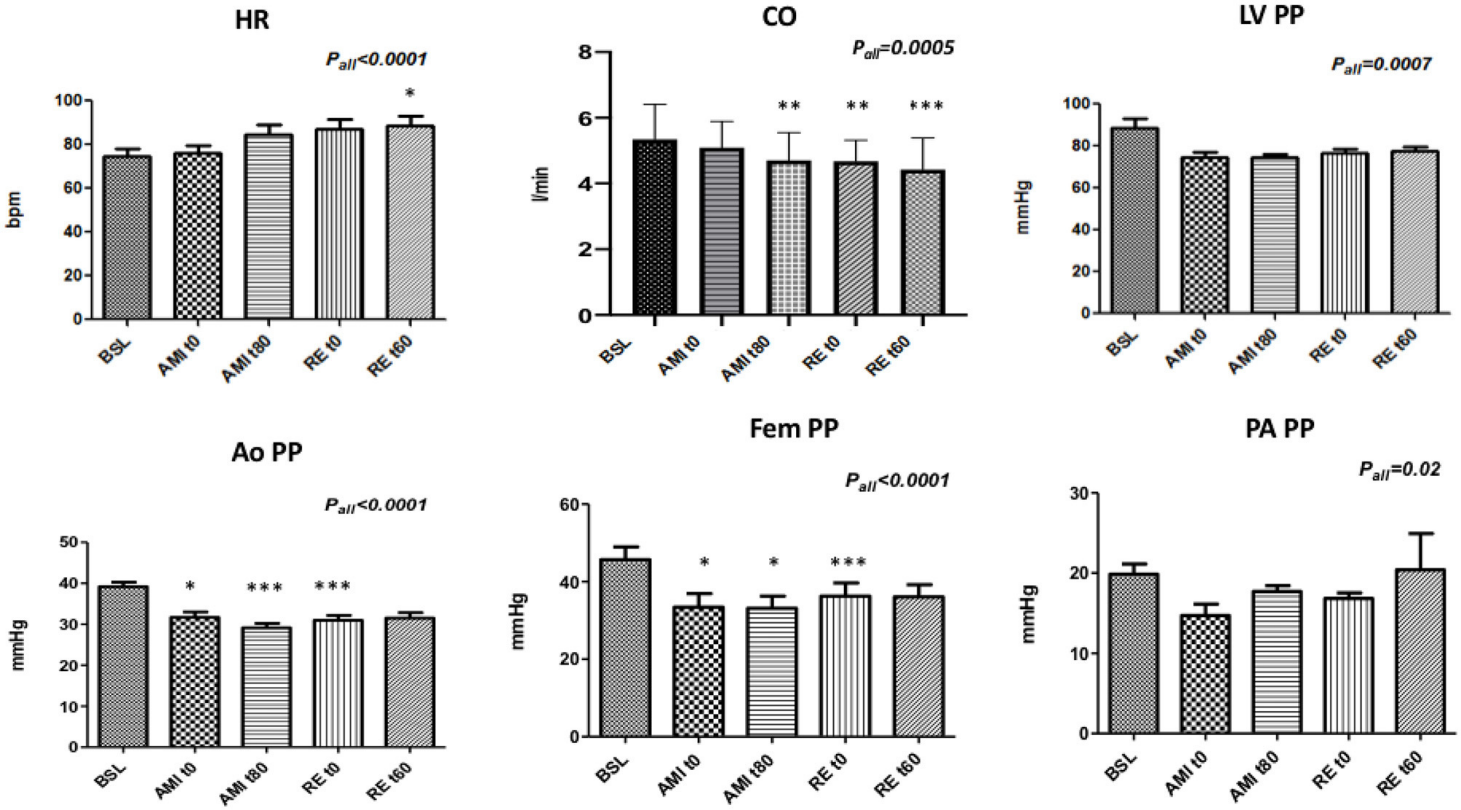
Modifications of HR, CO, and pulse pressure parameters during coronary sub-occlusion and reperfusion. HR, heart rate; CO, cardiac output; LV PP, pulse pressure of LV pressure; Ao PP, pulse aortic pressure; Fem PP, femoral pulse pressure; PA PP, pulse pressure of pulmonary artery pressure; BSL, baseline; AMI_t0−t80_, acute myocardial infarction at t0 and t80, respectively; RE_t0−t60_, reperfusion at t0 and t60, respectively. A generalized mixed model was used, with time as the fixed factor. The level of significance was set at 0.05. *p < 0.05; **p < 0.01; ***p < 0.0001. Data are presented as mean ± SEM for each variable (*N* = 17).

Figure 1 reports the modifications of pulse pressures of the same hemodynamic variables: LV pulse pressure (LV PP), aortic pulse pressure (Ao PP), femoral pulse pressure (Fem PP), and pulmonary artery pulse pressure (PA PP). The results are presented also in Supplementary Table 2.

HR increased by 19% from baseline to the end of reperfusion (*P*_ALL_ = 0.0001), while CO, systolic PLV, systolic PAo, and systolic Pfem decreased (*P*_ALL_ = 0.0005, *P*_ALL_ = 0.005, *P*_ALL_ = 0.005, and *P*_ALL_ < 0.0001, respectively).

According to a multiple-comparison analysis, the HR increased between AMI_t0_ and RE_t60_ (*p* = 0.02); CO dropped from 5.5 to 4.7 l/min from BSL to AMI_t80_ and RE_t0_ (both *p* = 0.002) and dropped further to 4.4 l/min at RE_t60_ compared to BSL (*p* < 0.0001); the systolic pressures of LV and aorta both dropped by 16 and 19%, respectively; between BSL and AMI_t0_ (*p* = 0.01 and *p* = 0.03, respectively) by 16 and 26%, respectively; between BSL and AMI_t80_ (*p* = 0.002 and *p* = 0.003, respectively) by 16 and 15%, respectively, between BSL and RE_t0_ (*p* = 0.001, *p* = 0.002, respectively), and by 16 and 15% between BSL and RE_t60_ (*p* = 0.003 and *p* = 0.036, respectively). The systolic femoral pressure dropped between BSL and AMI_t0_ (18%), AMI_t80_ (19%), and RE_t0_ (15%) (*p* < 0.0001, *p* = 0.004, and *p* < 0.0001, respectively).

When considering the pulse pressures of the above mentioned hemodynamic variables shown in Table 1, LV PP, Ao PP, and Fem PP decreased by 13, 20, and 21% from baseline to the end of reperfusion, respectively (*P*_ALL_ = 0.0007, *P*_ALL_ < 0.0001, and *P*_ALL_ < 0.0001, respectively). According to a multiple-comparison analysis, the Ao PP and the Fem PP dropped at AMI_t0_ (both *p* = 0.01), at AMI_t80_ (*p* < 0.0001 and *p* = 0.002, respectively), and at RE_t0_ (both *p* < 0.0001) compared to BSL. The modifications of pulse pressures during the LAD sub-occlusion and reperfusion are shown in Figure 1 and are reported in Supplementary Table 3.

**Figure 2:**
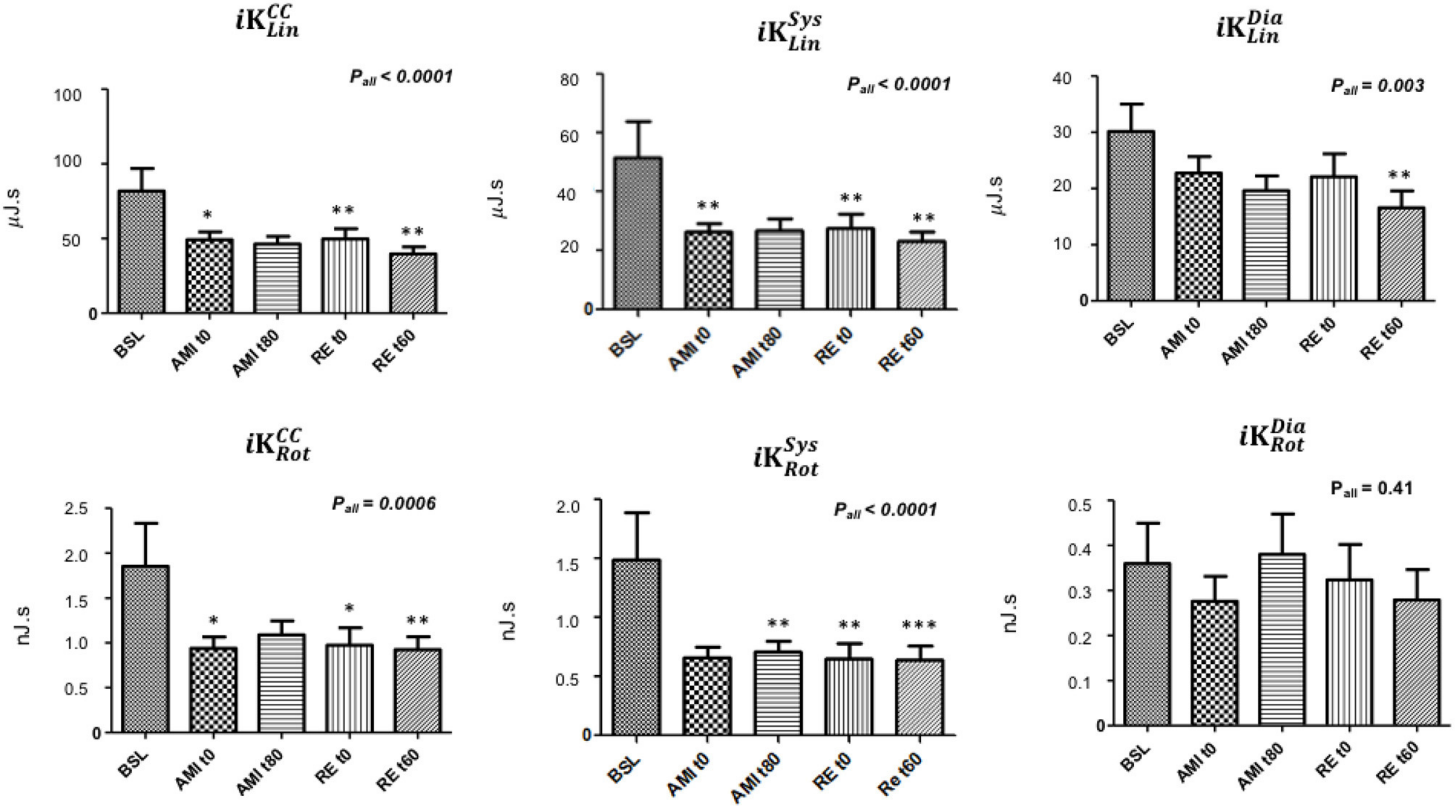
Modifications of parameters of *iK* during coronary sub-occlusion and reperfusion. ${iK}_{Lin}^{CC}$, ${iK}_{Lin}^{Sys}$, ${iK}_{Lin}^{Dia}$, *iK* of seismocardiography (SCG) in the linear dimension computed over the whole cardiac cycle (CC), the systolic phase (Sys), and the diastolic phase (Dia), respectively; ${iK}_{Rot}^{CC}$, ${iK}_{Rot}^{Sys}$, ${iK}_{Rot}^{Dia}$, *iK* of SCG in the rotational dimension computed over the whole CC, Sys, and Dia, respectively; BSL, baseline; AMI_t0−t80_, acute myocardial infarction at t0 and t80, respectively; RE_t0−t60_, reperfusion at t0 and t60, respectively. A generalized mixed model was used, with time as the fixed factor. The level of significance was set at 0.05. *p < 0.05; **p < 0.01; ***p < 0.0001. Data are presented as mean ± SEM for each variable (*N* = 17).

Figure 2 depicts the modifications of parameters of *iK* in the linear and rotational dimensions during the procedure.

All parameters of *iK*, except ${iK}_{Rot}^{Dia}$, decreased during LAD sub-occlusion and reperfusion. In the linear dimension, ${iK}_{Lin}^{CC}$, ${iK}_{Lin}^{Sys}$, and ${iK}_{Lin}^{Dia}$ dropped by 43, 52, and 53%, respectively (*P*_ALL_ < 0.0001, *P*_ALL_ <0.0001, and *P*_ALL_ = 0.03, respectively) from baseline to the end of reperfusion. In the rotational dimension, ${iK}_{Rot}^{CC}$ and ${iK}_{Rot}^{Sys}$ dropped by 30 and 38%, respectively (*P*_ALL_ = 0.0006 and *P*_ALL_ < 0.0001, respectively).

According to multiple comparisons, ${iK}_{Lin}^{CC}$ dropped by 20, 30, and 43% at AMI_t0_, RE_t0_, and RE_t60_, respectively, compared to BSL (*p* = 0.01, *p* = 0.007, and *p* = 0.0009, respectively); ${iK}_{Lin}^{Sys}$ dropped by 33, 45, and 52% at AMI_t0_, RE_t0_, and RE_t60_, respectively, compared to BSL (*p* = 0.003, *p* = 0.008, and *p* = 0.002, respectively); ${iK}_{Lin}^{Dia}$ dropped by 53% from BSL to RE_t60_ (*p* = 0.005). With regards to the rotational parameters of *i*K, ${iK}_{Rot}^{CC}$ dropped by 20, 30, and 30% at AMI_t0_, RE_t0_, and RE_t60_, respectively, compared to BSL (*p* = 0.01, *p* = 0.01, and *p* = 0.003, respectively); ${iK}_{Rot}^{Sys}$ dropped by 25, 38, and 38% from BSL to AMI_t80_, RE_t0_, and RE_t60_, respectively (*p* = 0.008, *p* = 0.003, and *p* < 0.0001, respectively).

**Figure 3:**
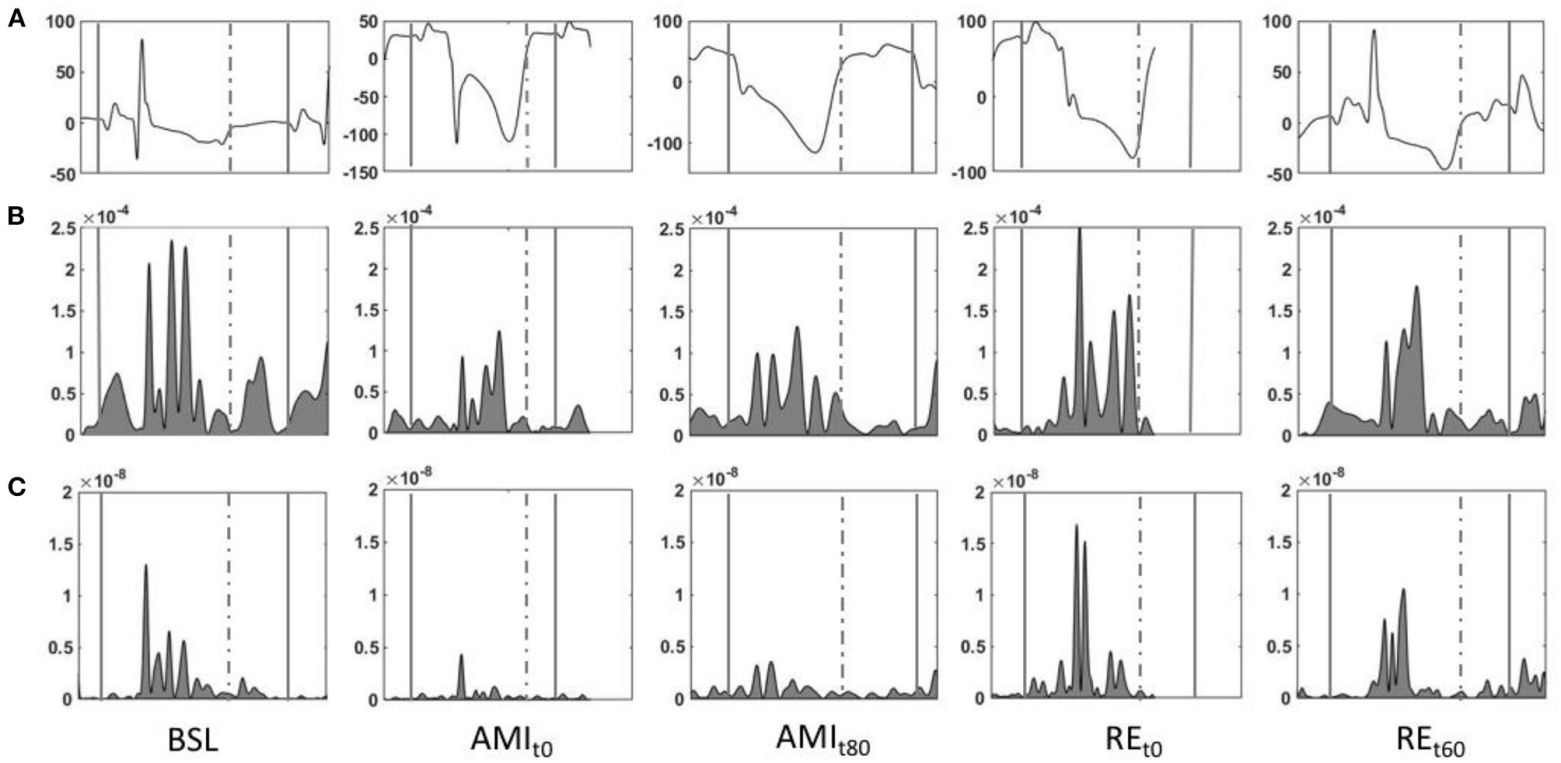
Representative figure showing the evolution of waveforms of *i*K computed from the seismocardiography (SCG) signals during the experimental procedure, specifically during baseline (BSL), AMI_t0_, AMI_t80_, RE_t0_, RE_t60_. The two solid lines [from the beginning of the P wave of beat *n* to the beginning of the P wave of beat *n* + 1 on the synchronous electrocardiogram (ECG)] denote the *i*K computed over the whole cardiac cycle (*i*K_CC_). The waveforms between the solid line and the dotted line (from the beginning of the P wave to the end of the T wave of beat *n* on the synchronous ECG) represent the *i*K of the systolic wave (*i*K_Sys_). The waveforms between the dotted line and the solid line (from the end of the T wave of beat *n* to the beginning of the P wave of beat *n* + 1 on the synchronous ECG) represent the *i*K of the diastolic phase (*i*K_Dia_). **(A)** ECG. **(B)** *i*K in the linear dimension. **(C)** *i*K in the rotational dimension. Linear **(B)** and rotational **(C)** *i*K drop at the onset of coronary sub-occlusion (AMI_t0_), compared to BSL, remains far below baseline values during the whole duration of AMI (AMI_t80_) and returns to normal level during reperfusion (RE_t0−t60_). BSL, baseline; AMI t0-t80, acute myocardial infarction at t0 and t80, respectively; RE t0-t60, reperfusion at t0 and t60, respectively; *i*K, integral of kinetic energy (J·s).

Figure 3 shows a representative case of modifications of *iK* during coronary occlusion and reperfusion for one animal.

Table 2 shows the generalized linear model used to associate pulse pressure parameters and CO with parameters of *iK*. All of the hemodynamic parameters, except PA PP, were significantly related to the parameters of *iK*, with a positive direction of association. LV PP was positively associated with ${iK}_{Lin}^{CC}$, ${iK}_{Lin}^{Sys}$, ${iK}_{Lin}^{Dia}$, ${iK}_{Rot}^{CC}$, and ${iK}_{Rot}^{Sys}$ (*p* < 0.0001, *p* < 0.0001, *p* = 0.03, *p* < 0.0001, and *p* < 0.0001, respectively); ${iK}_{Lin}^{CC}$, ${iK}_{Lin}^{Sys}$, ${iK}_{Rot}^{CC}$, and ${iK}_{Rot}^{Sys}$ were positively associated with the Ao PP (*p* < 0.001, *p* < 0.001, *p* = 0.008, and *p* = 0.001, respectively) and Fem PP (*p* = 0.01, *p* = 0.005, *p* = 0.05, and *p* = 0.01, respectively). Although these associations were positive and significant, they were still indirect, as shown by the too wide confidence intervals. The CO was also found to correlate with parameters of *i*K, especially with ${iK}_{Lin}^{CC}$, ${iK}_{Lin}^{Sys}$, ${iK}_{Rot}^{CC}$, and ${iK}_{Rot}^{Sys}$, with a positive direction of association (*p* = 0.002, *p* < 0.0001, *p* = 0.004, and *p* < 0.0001, respectively).

**Table 2:** Associations between pulse pressures and cardiac output with parameters of *i*K in the linear and rotational dimensions.

	**LV PP (mmHg)**			**Ao PP (mmHg)**			**Fem PP (mmHg)**			**PA PP (mmHg)**			**CO (L/min)**		
	**Coefficient**	**95% CI**	***p*-value**	**Coefficient**	**95% CI**	***p*-value**	**Coefficient**	**95% CI**	***p*-value**	**Coefficient**	**95% CI**	***p*-value**	**Coefficient**	**95% CI**	***p*-value**
SCG Lin CC (μJ·s)	0.14	0.07; 0.2	<0.0001	0.06	0.03; 0.09	<0.001	0.09	0.02; 0.15	0.01	0.002	−0.001; 0.005	0.29	8.1	3.0; 13.2	0.002
SCG Lin Sys (μJ·s)	0.02	0.08; 0.3	<0.0001	0.08	0.04; 0.11	<0.001	0.12	0.03; 0.19	0.005	0.002	−0.001; 0.006	0.16	11.7	5.6; 17.8	<0.0001
SCG Lin Dia (μJ·s)	0.2	0.02; 0.37	0.03	0.05	−0.04; 0.14	0.26	0.04	−0.14; 0.22	0.65	−0.001	−0.009; 0.007	0.78	2.2	−11.2; 15.6	0.75
SCG rot CC (nJ·s)	4.40	2.08; 6.72	<0.0001	1.49	0.4; 2.60	0.008	2.28	0.02; 4.53	0.05	0.06	−0.35; 15.2	0.22	2.5	0.8; 42.0	0.004
SCG Rot Sys (nJ·s)	5.56	2.89; 8.22	<0.0001	2.16	0.9; 3.37	0.001	3.32	0.7; 5.89	0.01	0.08	−0.03; 0.18	0.14	3.5	1.5; 5.4	<0.0001
SCG Rot Dia (nJ·s)	6.10	−3.03; 15.2	0.19	−3.36	−8.32; 1.60	0.18	−3.35	−13.3; 6.63	0.51	−0.08	−0.5; 0.36	0.72	−0.7	−7.8; 6.2	0.83

*iK SCG Lin-Rot, integral of kinetic energy of seismocardiography in the linear and rotational dimension, respectively; CC–Sys–Dia, cardiac cycle–systolic phase–diastolic phase; LV PP, left ventricle pulse pressure; Ao PP, aortic pulse pressure; Fem PP, femoral pulse pressure; PA PP, pulmonary artery pulse pressure*.

The parameters of *iK* have been associated to the Δ troponins and to the LVEF obtained at the end of the procedure (RE_t60_), but no significant associations were observed (Tables 3, 4, respectively).

**Table 3:** Spearman's correlation between delta troponins and parameters of *i*K.

***N* = 17**	***iK*_*cc*_**	***iK*_*Sys*_**	***iK*_*Dia*_**
**Linear dimension**			
Δ Troponins	*r* = −0.32, *p* = 0.23	*r* = −0.12, *p* = 0.66	*r* = −0.36, *p* = 0.2
**Rotational dimension**			
Δ Troponins	*r* = −0.17, *p* = 0.53	*r* = 0.06, *p* = 0.8	*r* = −0.16, *p* = 0.56

*iK_CC_, iK_Sys_, iK_Dia_: iK of seismocardiography computed over the whole cardiac cycle (CC), the systolic phase (Sys), and the diastolic phase (Dia), respectively*.

**Table 4:** Spearman's correlation between LVEF and parameters of *i*K.

***N* = 17**	***iK*_*cc*_**	***iK*_*Sys*_**	***iK*_*Dia*_**
**Linear dimension**			
LVEF	*r* = −0.24, *p* = 0.41	*r* = −0.48, *p* = 0.09	*r* = 0.10, *p* = 0.74
**Rotational dimension**			
LVEF	*r* = −0.30, *p* = 0.3	*r* = −0.30, *p* = 0.31	*r* = −0.43, *p* = 0.13

*iK*_*CC*_, *iK*_*Sys*_, *iK*_*Dia*_, *iK* *of seismocardiography computed over the whole cardiac cycle (CC), the systolic phase (Sys), and the diastolic phase (Dia), respectively; LVEF, left ventricle ejection fraction*.

## Discussion

We reported for the first time the direct evidence that non-invasive, multi-dimensional SCG can quantify the cardiac kinetic energy and continuously track its changes during AMI and reperfusion in a closed chest swine model of AMI. We have previously highlighted the potential of micro-accelerations and gyroscopes in providing reliable information on the contractility status of the heart (2, 30): as found in previous study, metrics of *iK* are able to follow changes in cardiac contractility with high accuracy and were related to SV and CO (2); the increased cardiac kinetic energy measured with micro-accelerometers and gyroscopes was directly related to the rise of sympathetic nerve traffic during an end-voluntary maximal apnea (31); signals acquired with multi-dimensional SCG and BCG could monitor cardiac deconditioning in astronauts during simulated microgravity (32). With the present research, we demonstrate, for the first time, that the cardiac kinetic energy recorded with multi-dimensional non-invasive SCG, along with hemodynamic and echocardiographic findings, drops during AMI compared to normal cardiac inotropic state and does not improve during coronary reperfusion (33), likely reflecting a reduced left ventricular function of ischemic origin and further confirmed by the rise of plasma troponin levels and the drop of LVEF associated with regional LV wall abnormalities, which persisted despite revascularization. After sudden coronary artery occlusion, the unsupplied myocardium loses its ability to shorten and lengthen, and myocardial contractile function drastically drops (34). With relief of ischemia and reestablishment of coronary blood flow, there is a persistent wall motion abnormality despite reperfusion and viable myocytes (35). The sudden drop of *iK* observed immediately after coronary occlusion likely reflects the ischemic dysfunction due to supply lost, and the persistent drop of *iK* during reperfusion likely reflects further the myocardial reperfusion injury (33, 35). These conclusions are further supported by the drop of LVEF and the rise of plasma troponins at the end of revascularization and further corroborated by modifications of hemodynamic parameters showing the same trend of *i*K parameters during the experimental AMI.

Acute activation of sympathetic nervous system following AMI has been previously described in several investigations (36): the acute surge of catecholamines observed during prolonged acute myocardial ischemia (longer than 10 min at least) can reach plasma concentrations as high as 1,000 times of normal plasma levels, especially in cardiogenic shock (37), and such high concentrations are cardiotoxic, potentially inducing myocardial necrosis (38), with myocardial detrimental effect (37). Thus, the cardiotoxic effect of catecholamines secondary to sympathetic overactivity may be evoked as an additional mechanism contributing to the persistent drop of LVEF and *i*K parameters after reperfusion.

Previous authors extensively investigated the utility of micro-accelerometers and gyroscopes as diagnostic tools for acute ischemic myocardial impairment (7–9), and results are all in favor to suggest the potential of micro-accelerometers and gyroscopes in the early detection of myocardial dysfunction of ischemic origin. Backer et al. used the SCG to detect myocardial impairment on nine swine and differentiate ischemia from hypovolemia as causes of myocardial dysfunction (7); Elle and colleagues used a three-axes accelerometer sensor on three anesthetized swine to recognize regional myocardial ischemia early following LAD surgical occlusion and found that the acceleration signals dropped by 40% at only 130 s after coronary occlusion; Halvorsen et al. operated on 14 anesthetized swine a LAD surgical occlusion for 60 s while recording the velocities of LV wall with a three-axes accelerometer and reported that myocardial wall regional impairment is accompanied by concurrent changes in accelerometer velocities both during systole and relaxation (9). They further demonstrated the potential of accelerometers in the detection of myocardial ischemia in patients undergoing cardiac surgery (13).

The present investigation strongly reinforces and complements the previous ones by adding several novelties. First, we used three-axial sensors in three cardinal axes provided with linear and rotational channels to obtain a multi-dimensional assessment of blood flow and cardiac function with six degrees of freedom. Second, we applied Newtonian equations on acceleration signals to compute the scalar parameters of kinetic energy and its temporal integral *iK* for each contractile cycle in order to quantitatively measure the cardiac kinetic energy produced during a contractile cycle as well as during the systolic and diastolic phases (2, 11, 39). Third, we demonstrated that the fall of cardiac *iK* following LAD sub-occlusion is maintained for the whole duration of the coronary occlusion and does not improve during reperfusion. The fall of *i*K parameters is likely of ischemic origin as suggested by the rise of plasma troponins and by the drop of LVEF along with regional LV wall abnormalities, which persist at the end of the experimental procedure. Fourth, changes of *iK* parameters during the whole procedure were positively correlated with changes of invasive pulse pressures and CO, which fell as well during acute myocardial infarction, showing the same evolution pattern of *i*K parameters. Fifth, the drop of *iK* observed during occlusion and reperfusion was not related to the infarct size as estimated by early troponins release nor to the severity of myocardial contraction as estimated by the LVEF. Sixth, associations between *i*K and invasive pulse pressures are observed only with left-side pulse pressures, that is, LV PP, Ao PP, and Fem PP, but not with PA PP. Seventh, we used a closed-chest porcine model of AMI, which represents a valuable and suitable surrogate for myocardial infarction in humans (40). Reduction by 60% of coronary blood flow, induced by using a coronary balloon, was enough to trigger electrical, metabolic, and mechanical modifications of cardiac function as demonstrated by ST segment abnormalities, the rise of cardiac troponins, and the drop of LVEF along with regional LV wall abnormalities. Additionally, the sample size accounted for 17 out of 21 pigs that is far larger compared to previous investigations (5, 8, 9, 13). This observation makes the authors believe that, in this very context of experimental AMI with acute LV regional dysfunction and no concomitant AMI-related heart valve disease, linear and rotational *i*K parameters, in particular systolic ones, provide reliable information on LV contractile dysfunction and its effects on the downstream circulation.

As explained above, the automatic identification of P, Q, R, S, and T waves on the ECG allowed for the identification of the cardiac cycle on the SCG waveforms. By combining these reference points, the systolic and diastolic phases can also be identified (12). The present investigation reported also the different impact of AMI on systolic and diastolic SCG waveforms. Indeed while the *iK* during the systolic phase dropped during coronary sub-occlusion and reperfusion both in linear and rotational dimensions, the *iK* during the diastolic phase seems to be less influenced by the ischemic event, showing a modest significant drop in the linear dimension and no changes at all in the rotational dimension. This makes the authors believe that an acute ischemic cardiac event with predominant systolic dysfunction has a deep impact mainly on the systolic SCG waveforms rather than the diastolic ones. The authors speculate that the diastolic component of *i*K may reflect more the filling functions of the LV rather than its contractile properties. To further corroborate this viewpoint, the diastolic *iK* was not associated with any of the pulse pressure parameters nor the CO, except the linear diastolic *i*K with the LV PP, but with a weak significance.

Even though critical differences exist between our technique and those used by our predecessors (7–9, 13, 14) (i.e., non-intrusive device, remotely controlled system, automatic analysis, use of linear and rotational channels, computation of scalar parameters from acceleration signals), our observations further confirm the core concept that micro-accelerometers and gyroscopes can reliably monitor cardiovascular changes occurring during AMI and reperfusion.

Spaccarotella et al. have recently demonstrated that smartwatches ECG can detect ST segment elevation and depression with high sensitivity and specificity compared to a standard 12-lead ECG, and this might empower the earlier detection of ECG abnormalities in patients with acute coronary syndrome (41).

We presented a device capable of computing the integral of kinetic energy of a contractile cycle recorded with micro-accelerometers and gyroscopes, with the aim to provide information on the mechanical activity of the heart. We previously demonstrated the capability of this device to follow changes of cardiac contractility in different conditions as already mentioned (2, 12, 31, 32, 42). With the present investigation, we add that this device can detect an acutely failing heart of ischemic origin by providing a parameter of kinetic energy. An important possible application of this renewed technology is the follow-up of patients with myocardial dysfunction in the mid-long term after an acute ischemic event. Thanks to the easy-to-use properties of the device, cardiac patients might be empowered to follow their own medical conditions, as it is already the case with atrial fibrillation diagnosed with smartwatches. To our knowledge, markers of myocardial mechanical function are not provided by the smartwatches currently in use, and we believe that this device may complement the existing ones by adding the cardiac kinetic energy as a new parameter of myocardial mechanical function and thus may prove useful to track changes in myocardial mechanical activity of heart failure patients in the near future.

Of course, this device must not be considered as a competitor to traditional standards and guidelines universally used for cardiac patient's follow-up but as a complement to them.

### Limitations

Some limitations need considerations. Because of marked differences in anatomy, heart, and vessel orientation, the effects of myocardial infraction in humans are likely to differ in the three axes investigated in this study, but the observations on the *iK* parameters which include the three axes should remain valid. We also cannot report on the effects of AMI on multidimensional BCG in this study because of marked differences in body mass distribution between the experimental animal model that we investigated and the human beings to which the original prototype was made for (2, 11).

Indeed because of technical limitations during the experimental procedure, mainly the recumbent position of the animal, the BCG sensor was placed externally to the left iliac crest and not close to the body center of mass (lower back of the animal) as recommended (2, 11). Placing the BCG module in this wrong position means that the recorded signals cannot be considered as BCG ones. The authors were not able to place this module over the lumbar lordosis curve for the following reasons: the recumbent position of the animal and the consequent difficulties to place the device under it and the difficulty to access this region and to promptly remove the device whenever a cardiac arrest for ventricular arrythmias occurred and prompt defibrillation was required. Indeed whenever resuscitation was required, the device was promptly removed, and easy accessibility to it was mandatory for the sake of the safety of the operators and the animal.

Despite the fact that this technique has not been standardized yet with large-scale-based studies so that no normal values of kinetic energy can be provided, this limitation was encompassed with the repeated-measures study design, where each animal was its own control. The same study design was adopted in our previous works (2, 12, 31, 32, 42).

With regard to the experimental procedure, some readers may arise concerns that the observed cardiovascular modifications might be due to general anesthesia, specifically to sevoflurane (43) and azaperone (44). However, the authors are confident to conclude that the cardiovascular modifications occurring during the experimental procedure were likely attributable to acute myocardial ischemia and not to general anesthesia for several reasons: first, the drop by ±5 mmHg of the mean arterial pressure observed in the sham group cannot explain the large reduction in the mean systemic blood pressure that we observed during myocardial infarction, and this allowed the authors to rule out the depressant effect of general anesthesia on the hemodynamic parameters; second, sevoflurane has higher hemodynamic stability and fewer arrhythmic events compared to other volatile agents (24), and sevoflurane inhalation was within normal range (1.8 to 2.5% of MAC); third, since azaperone has a duration of action of 2 to 3 h in young pigs with a peak within the first 30 min (45) and since the procedure was started after 4 h at least of steady state, the effects of this drug on systemic circulation cannot be considered as responsible for the observed hemodynamic impairment after AMI; and fourth, the rise of troponin levels and the drop of LVEF associated with regional LV wall abnormalities which persist after reperfusion are all in favor to suggest that hemodynamic impairment and reduction of *i*K parameters were a direct consequence of acute myocardial ischemia and not of anesthetic agents.

We did not find any change in the left ventricle diastolic pressure during the experimental procedure. The authors attribute this phenomenon to the effect of the mechanical ventilation with a positive end-expiratory pressure of 5 cm H_2_O, which induces a fall in transpulmonary flow and thus in the venous return to the LV, with the global effect of reducing the LV preload (46).

The study design was conceived to induce a cardiogenic shock. Since in swine only 25% of LV mass is supplied by the right coronary artery and 25% by the left circumflex artery, occluding these arteries would have probably not induced a cardiogenic shock. Further studies should be designed to assess the consequences of less extensive MI on SCG signals.

The observational period after reperfusion is relatively short; however, the study design was initially conceived to determine whether and how acute myocardial infarction and reperfusion affect the SCG signals and the derived parameters with no additional observational period. The positive and encouraging results obtained with this pivotal study set another step toward the validation of this renewed technique in the context of acute coronary diseases and undoubtedly justify further research on the long-term effect of MI on SCG signals.

Despite the limitations described above, this study further reinforces the need to investigate on the utility of micro-accelerations and gyroscopes in the detection of acute myocardial infarction on patients in real life and their potential as monitoring tools for the assessment of cardiovascular function following an acute ischemic cardiac event.

## Conclusions

To our knowledge, this is the first study to demonstrate the potential of non-intrusive, multi-dimensional SCG to monitor in real time the functional status of cardiac muscle during AMI with predominant systolic dysfunction followed by coronary reperfusion and to provide a quantitative assessment of cardiac kinetic energy computed from acceleration signals. Thanks to its easy-to-use properties, this automatic and remotely controlled system may empower healthcare providers and patients to monitor cardiovascular status in real life and may help to remotely detect any cardiac functional abnormalities early. Of course, this device must not be considered as a competitor to traditional standards and guidelines universally used for a cardiac patient's follow-up but as a complement to them.

## Data Availability Statement

The raw data supporting the conclusions of this article will be made available by the authors, upon reasonable request.

## Ethics Statement

The animal study was reviewed and approved by Institutional Ethics Committee on Animal Welfare from the Faculty of Medicine from the Université Libre de Bruxelles (ULB, Brussels, Belgium) (Acceptation Number: 654N).

## Author Contributions

PvdB and SM conceived the idea and the design of the study. LP designed the animal model of AMI and carried out the whole experimental procedure supported by FS and obtained all the invasive hemodynamic parameters data. AHo provided the technical support and designed the specific Toolbox in MathLab for the correct extrapolation of all metrics from SCG. SM had full access to all data in the present investigation, extrapolated all SCG data, and takes responsibility for the integrity of the data and the accuracy of the data analysis. AHe was responsible for the experimental procedure on the sham group and provided the results for the sham group. JRac performed statistical analysis. SM and LP drafted the manuscript. All authors revised the manuscript critically for important intellectual content, proofread, and made corrections to this manuscript.

## Conflict of Interest

P-FM, DG, and AHo declare having direct ownership of shares in Healthcare Company. The remaining authors declare that the research was conducted in the absence of any commercial or financial relationships that could be construed as a potential conflict of interest.
