# Supplementary material for: Quantification of Cardiac Kinetic Energy and Its Changes During Transmural Myocardial Infarction Assessed by Multi-Dimensional Seismocardiography
Source: Front Cardiovasc Med. 2021 Mar 8;8:603319. doi: 10.3389/fcvm.2021.603319 (PMC7982421; doi:10.3389/fcvm.2021.603319)
Supplement: Supplementary file 1 [file Data_Sheet_1.docx]

| **Table 1S.** | | **Swine** | **HR** | **Fem SAP** | **Fem DAP** | **Fem MAP** | **PAPs** | **PAPd** |
| --- | --- | --- | --- | --- | --- | --- | --- | --- |
| **Sham** | **BSL** | **1** | 80 | 106 | 52 | 73 | 30 | 15 |
|  |  | **2** | 70 | 110 | 54 | 75 | 31 | 16 |
|  |  | **3** | 73 | 100 | 43 | 62 | 27 | 12 |
|  |  | **Mean ± SD** | **74 ± 5** | **105 ± 5** | **50 ± 6** | **70 ± 7** | **29 ± 2** | **14 ± 2** |
|  | **T1** | **1** | 93 | 93 | 45 | 62 | 33 | 15 |
|  |  | **2** | 79 | 98 | 46 | 69 | 31 | 16 |
|  |  | **3** | 71 | 99 | 43 | 63 | 28 | 13 |
|  |  | **Mean ± SD** | **81 ± 11** | **97 ± 3** | **45 ± 2** | **65 ± 4** | **31 ± 3** | **15 ± 2** |
|  | **T2** | **1** | 103 | 96 | 46 | 66 | 31 | 15 |
|  |  | **2** | 76 | 98 | 45 | 64 | 31 | 17 |
|  |  | **3** | 79 | 100 | 41 | 61 | 29 | 13 |
|  |  | **Mean ± SD** | **86 ± 15** | **98 ± 2** | **44 ± 3** | **64 ± 3** | **30 ± 1** | **15 ± 2** |
| Results from a sham group composed of three swine from a previous investigation. HR, heart rate; Fem SAP, femoral systolic arterial pressure; Fem DAP, femoral dastolic arterial pressure; Fem MAP, femoral mean arterial pressure; PAPs, systolic pulmonary artery pressure; PAPd, diastolic pulmonary artery pressure. | | | | | | | | |
|  |  |  |  |  |  |  |  |  |
|  |  |  |  |  |  |  |  |  |
|  |  |  |  |  |  |  |  |  |

| **Table 2S. Modifications of LV, aortic, femoral and pulmonary pulse pressures during LAD occlusion and reperfusion.** | | | | |
| --- | --- | --- | --- | --- |
|  |  |  |  |  |
|  |  |  |  |  |
| **Time** | **LV PP (mmHg)** | **Ao PP (mmHg)** | **Fem PP (mmHg)** | **PA PP (mmHg)** |
|  |  |  |  |  |
| **BSL** | 88.6 ± 16.5 | 39.2 ± 4.0 | 45.7 ± 1.9 | 18.6 [17.6; 22.8] |
|  |  |  |  |  |
| **AMI t0** | 74.8 ± 9.1 | 31.7 ± 4.95* | 33.4 ± 13.0* | 15.5 [10.8; 18.0] |
|  |  |  |  |  |
| **AMI t80** | 74.8 ± 6.0 | 29.1 ± 4.3‡ | 33.1 ± 12.2* | 17.7 [16.3; 19.5] |
|  |  |  |  |  |
| **RE t0** | 76.5 ± 7.5 | 31.0 ± 4.5‡ | 36.3 ± 12.8‡ | 16.5 [15.1; 19.6] |
|  |  |  |  |  |
| **RE t60** | 77.4 ± 8.4 | 31.5 ± 4.5 | 36.1 ± 11.4 | 18.1 [14.4; 19.6] |
|  |  |  |  |  |
|  | | | | |
| ***P_ALL_-value*** | ***0.0007*** | ***<0.0001*** | ***<0.0001*** | ***0.02*** |
|  |  |  |  |  |
| BSL: baseline; AMI t0-t80: acute myocardial infarction at t0 and t80, respectively; RE t0-t60: reperfusion, t0 and t60, respectively. HR: heart rate; LV PP, LV pulse pressure; Ao PP, aortic pulse pressure; Fem PP, femoral pulse pressure; PA PP, pulmonary artery pulse pressure. Results from multiple comparison analysis account for comparison of the different timepoints against BSL. *: p<0.05; †: p<0.01; ‡: p<0.0001. Data are presented as mean ± SD or median [P25; P75] according to data distribution. | | | | |
|  |  |  |  |  |
|  |  |  |  |  |
|  |  |  |  |  |
|  |  |  |  |  |

| **Table 3S. Modification of *i*K of SCG during LAD occlusion and reperfusion.** | | | | | | |
| --- | --- | --- | --- | --- | --- | --- |
|  |  |  |  |  |  |  |
| **Time** | ${\boldsymbol{i}\mathbf{K}}_{\boldsymbol{Lin}}^{\boldsymbol{CC}}$ **(𝜇J.s)** | ${\boldsymbol{i}\mathbf{K}}_{\boldsymbol{Lin}}^{\boldsymbol{Sys}}$ **(𝜇J.s)** | ${\boldsymbol{i}\mathbf{K}}_{\boldsymbol{Lin}}^{\boldsymbol{Dia}}$ **(𝜇J.s)** | ${\boldsymbol{i}\mathbf{K}}_{\boldsymbol{Rot}}^{\boldsymbol{CC}}$ **(nJ.s)** | ${\boldsymbol{i}\mathbf{K}}_{\boldsymbol{Rot}}^{\boldsymbol{Sys}}$ **(nJ.s)** | ${\boldsymbol{i}\mathbf{K}}_{\boldsymbol{Rot}}^{\boldsymbol{Dia}}$ **(nJ.s)** |
|  |  |  |  |  |  |  |
| **BSL** | 61.7 [42.1; 102.4] | 40.2 [25.1; 59.0] | 27.6 [15.4; 42.6] | 1.0 [0.9; 1.9 ] | 0.8 [0.7; 1.5] | 0.2 [0.2; 0.4] |
|  |  |  |  |  |  |  |
| **AMI t0** | 49.6 [35.9; 66.8]* | 27.0 [18.1; 35.1]† | 20.1 [14.9; 32.9] | 0.8 [0.5; 1.3]* | 0.7 [0.3; 0.9] | 0.2 [0.1; 0.4] |
|  |  |  |  |  |  |  |
| **AMI t80** | 42.6 [28.7; 62.1] | 23.7 [12.6; 34.6] | 15.8 [11.2; 26.1] | 0.9 [0.7; 1.4] | 0.6 [0.4; 0.8]† | 0.3 [0.1; 0.5] |
|  |  |  |  |  |  |  |
| **RE t0** | 43.0 [29.7; 65.0]† | 22.2 [16.3; 33.3]† | 16.7 [11.2; 27.2] | 0.7 [0.5; 1.3]* | 0.5 [0.3; 0.8]† | 0.2 [0.1; 0.4] |
|  |  |  |  |  |  |  |
| **RE t60** | 35.0 [25.9; 63.8]† | 19.3 [15.0; 27.0]† | 13.0 [8.7; 19.3]† | 0.7 [0.6; 1.4]† | 0.5 [0.3; 0.8]‡ | 0.2 [0.1; 0.3] |
|  |  |  |  |  |  |  |
|  | | | | | | |
| ***P_ALL_-value*** | ***<0.0001*** | ***<0.0001*** | ***0.03*** | ***0.0006*** | ***<0.0001*** | 0.41 |
|  |  |  |  |  |  |  |
| BSL: baseline; AMI t0-t80: acute myocardial infarction at t0 and t80, respectively; RE t0-t60: reperfusion, t0 and t60, respectively; iK SCG Lin-Rot: integral of kinetic energy of seismocardiography in the linear and rotational dimension, respectively; CC-Sys-Dia: cardiac cycle, systolic phase, diastolic phase, respectively. Results from multiple comparison analysis account for comparison of the different timepoints against BSL. Data are presented as median [P25; P75]. *: p<0.05; †: p<0.01; ‡: p<0.0001. | | | | | | |
|  |  |  |  |  |  |  |
|  |  |  |  |  |  |  |
